# Parsing cultural impacts on regret and risk in Iran, China and the United Kingdom

**DOI:** 10.1038/s41598-018-30680-7

**Published:** 2018-09-14

**Authors:** Li Li, Shiro Kumano, Anita Keshmirian, Bahador Bahrami, Jian Li, Nicholas D. Wright

**Affiliations:** 1 School of psychological and cognitive sciences and Beijing Key Laboratory of Behavior and Mental Health, Peking University, Beijing, China; 2 Institute of Cognitive Neuroscience, University College London, London, UK; 3 NTT Communication Science Laboratories, NTT Corporation, Kanagawa, Japan; 4 Department of Psychology and Education, University of Tehran, Tehran, Iran.University of Tehran, Jalal Al-e-Ahmad Avenue, Tehran, Iran; 5 Department of Cognitive Science, Institute for Cognitive Science Studies (ICSS), Pazhouheshkadeh Blvd, Safir Omid Blvd, 4th Phase, Pardis New City, Pardis, Iran; 6 PKU-IDG/McGovern Institute for Brain Research, Peking University, Beijing, China; 7 Pellegrino Center for Clinical Bioethics, Georgetown University Medical Centre, Washington, USA; 8 Intelligent Biology Ltd, London, UK; 9 Faculty of Psychology and Educational Sciences, Ludwig Maximilian University, Leopoldstrasse 13, 80802, Munich, Germany

## Abstract

Value-based choices are influenced both by powerful counterfactuals, such as regret, and also by risk in potential outcomes. Culture can profoundly affect how humans perceive and act in the world, but it remains unknown how regret in value-based choice and key aspects of risk-taking may differ between cultures. Here our computational approach provides precise and independent metrics, grounded in extensive neurobiological evidence, for the influences of risk and regret on choice. We test for commonalities and differences across three diverse cultures: Iran, China and the UK. Including Iran matters because cross-cultural work on value-based choice is lacking for this key 21^st^ Century culture, and also because patterns across the three cultures arbitrates between explanations for differences. We find commonalities, with regret influencing choice across cultures and no consistent cultural difference seen. However, for risk, unlike in both Chinese and Westerners’ choices, Iranians are risk-seeking – findings consistent across two task variants and further explained by Iranians showing less subjective impact of negative, but not positive, outcomes of risky choices. Our computational approach dissects cultural impacts on two key neurobiologically-grounded quantities in value-based choice, showing that neuroscientific accounts cannot a priori isolate such quantities from culture in the cognitive processes underlying choice.

## Introduction

Regret and risk both powerfully drive how humans choose between options based on their potential rewards and punishments. Regret is an example of counterfactual thinking in which we would have been better off if we had chosen differently, and it has been quantified computationally within neurobiological accounts of choice^1,2^. Risk is central to value-based choice across fields ranging from financial economics^3^ to animal foraging^4^, and again precise metrics have quantified risk in decision neuroscience^5–8^. Thus, considerable work has characterised such modulators of value-based choice in laboratory experiments primarily in the West, which have been prominent in accounts of choice based in neural computations^9^, in which computational approaches also enable the impacts of distinct value modulators to be parsed^5,6^. Considerable work, however, also suggests culture may profoundly affect how humans perceive, understand and act in the world^10–13^. But commonalities and differences in the impact of regret between cultures in value-based choice are unknown. So too are key aspects of how culture may affect risk-taking. Therefore, here we employ computational modelling to isolate and examine the impacts of risk and regret on individuals’ value-based choices in three diverse cultures – Iran, China and the UK.

Despite considerable interest in the impact of counterfactuals such as regret on value-based choice^1,2^, primarily in the West, we report the first laboratory experiments directly comparing cultures. This is motivated by previous work using recall of autobiographical life events, largely comparing the West and East Asia, suggesting differences in the nature of regrets between cultures^14,15^. Counterfactual thinking also relates directly to influential cultural theory in which Westerners show greater agency over their choices than East Asians^10^, so counterfactuals or feedback may have a greater impact. Our task in which participants choose between financial options and our model-based analysis help mitigate linguistic challenges cross-culturally. For example it is debated whether Dutch findings on *spijt* relate to English findings on “regret”^16,17^. Our methods contribute to emerging attempts to develop more implicit rather than explicit methods for cross-cultural research^18^.

However, it is also critical to isolate potential cultural impacts on regret from those on another key modulator of value-based choice: risk. Considerable primarily Western work has shown that computational modelling of tasks using trial-by-trial metrics of risk derived from financial economics (e.g. variance or standard deviation) can reliably capture the impact of risk on an individual’s choices^7^ and isolate them from other value modulators, such as outcome valence, both behaviourally and neutrally^5,6,19^. Using such techniques here to robustly assay a specific aspect of risk is important given previously inconsistent findings in cross-cultural studies of risk, particularly comparing East Asia and the West, which used small numbers of decisions with risk more broadly operationalised. While two well-known studies reported greater Chinese than US risk-taking^20,21^, one study replicated that finding^22^, one only found greater Chinese than UK risk-taking with one of the two analytic measures employed^23^, and three studies found no difference in risk-taking between U.S. and East Asian samples^24–26^. In addition to this previous heterogeneity, given the challenge of the “replication crisis” in psychology more broadly^27^, we thus also examine two variants of our task using separate participant groups for each task in each culture.

Finally, while much of the work above focusses on East Asia and the West, the inclusion of Iran is important here to interpret and robustly identify cultural commonalities and differences. We study Iran for two reasons. Firstly, Iran is a key specific case. It is a key Twenty First Century actor with a highly distinctive cultural, linguistic, religious and political tradition and geopolitical significance. However, we are aware of only one experimental study comparing any aspect of value-based choice to other countries, which looked at trends across six countries in a public-goods dilemma^28,29^. Very few such studies exist within Iran.

Secondly, including Iran provides methodological traction to interpret cultural effects^30^. Iran and China compared to the UK share key attributes that may explain cultural differences, for example both are developing countries. To the Eurocentric observer, both may also be considered collectivist rather than individualist^31^. This latter observation is important as a prominent explanation for potentially greater Chinese than Western risk-taking is the ‘cushion hypothesis’, whereby people in collectivist societies more likely receive financial help when in need and so are less risk averse than people in individualistic societies like the UK^21^. However, other reasons suggest instead that Iran may differ from China and the UK. Risk preference measured in 76 countries using surveys hints at increased preference for risk in Iran relative to China and the UK, although it did not compare specific countries^32^. Value-based choice in a public goods game showed almost identical behaviour in China and the UK that differed markedly to that in Muscat, Riyadh and Istanbul that are geographically proximal to Iran, and which was attributed to cultural norms and institutions^33^. Unlike those in the UK and China, the Iranian population has more recently been involved unpredictably in war and military service, with such factors in other countries shown to affect value-based choice^34^. Iranian institutions may also reflect and shape attitudes to risk and return, for example with parts of its banking system governed by Sharia law since 1983^35^.

**Figure 1:**
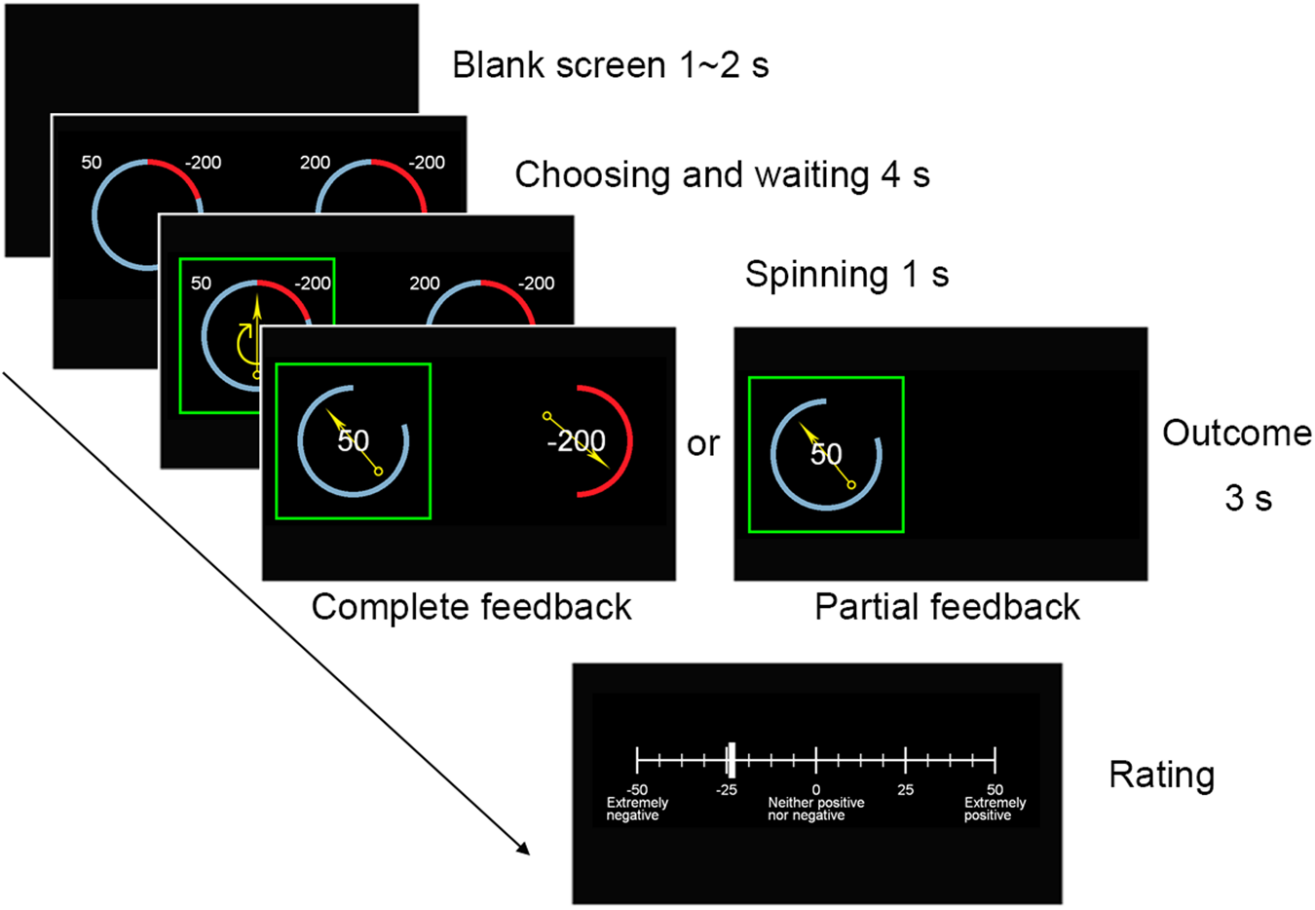
Experimental design. In each trial, individuals first evaluated two different lotteries with different potential outcomes and probabilities. They had to choose one within 4 s. After choosing, a green square highlighted the selected lottery. After the 4 s, an arrow appeared in the centre of the selected lottery and span for 1 s, with its final location indicating the trial’s outcome. Participants then subjectively rated their feelings about that outcome on an axis from −50 (extremely negative) to 50 (extremely positive). To separately assay potential effects of the lottery outcomes on subjective ratings, we used two feedback conditions. In the ‘complete-feedback’ condition participants saw both lotteries’ outcomes in the trial, so they may feel regret (or relief) by witnessing the unchosen lottery’s outcome. In the ‘partial-feedback’ condition they only saw the chosen lottery’s outcome, which may elicit disappointment (or joy) about the unrealized outcome in the chosen gamble. Our two task variants (Exp1 and Exp2) differed in two ways. First, the stimulus list differed. Exp1 used a stimulus list used previously^37^, which is potentially limited as regressors of interest are highly correlated across the list (Table 2), and further as the large EV differences between lottery pairs in most trials may dominate subtle cultural effects on risk or anticipated regret. Thus Exp2 used smaller EV differences within trials and less correlated regressors of interest across the set (details in Table 3 and S.3–S.6). Secondly, because previous work suggested cultural differences whereby initial trials in blocks affect Westerners more than Chinese:^45^ Exp1 presented partial- and complete-feedback conditions in 12 trial blocks; and Exp2 presented trials in random mixed order throughout.

To isolate the impact of culture on two key modulators of value-based choice—regret and risk—we exploit a task and computational analysis that assays each of these components independently. In our task, individuals must choose between two financial gambles, then receive feedback and make a subjective rating (Fig. 1). To enhance our findings’ robustness we look across two variants of our task (Fig. 1) and for convergent evidence across three measures: choice behaviour; subjective reports; and reaction times (RTs). We predicted both risk and regret would affect the process of value-based choice in all three cultures, but were agnostic as to whether cultural impacts would affect Iran and China relative to the UK, or China and the UK relative to Iran.

## Results

We tested the effects of risk and regret on financial decision-making using a ‘wheel of fortune’^36,37^ task (Fig. 1) in three distinct countries: China, Iran and the UK. Given the challenges of replicability we also tested two variants of the task, each in a new sample in each culture. We thus tested 117 participants overall, with six groups of participants in a 3 culture × 2 task variant (‘Exp1’ and ‘Exp2’) design.

### Regret and risk affect choice in all three cultures

Initially, we sought to isolate impacts of risk and regret on choice behaviour. We first conducted model comparison on choice behaviour in each of the six groups separately. The winning model in all six datasets included the anticipated regret (R) parameter, as shown in Table 1; with the summed BIC for the EV + SD + R model ≥ 16.4 smaller than the EV + SD model in all six datasets, and the EV + D + R ≥ 6.0 smaller than the EV + D in all six datasets. With respect to risk (SD), the winning model included SD in five of the six datasets: the EV + SD + R model won in both Iranian and both UK datasets, while in one Chinese dataset the EV + SD + D + R model won and in only one Chinese dataset did the EV + D + R model without risk win. Analysing the two Chinese datasets together, the tied winning models both included risk and regret (BIC EV + SD + R model = 2,080.7; EV + SD + D + R 2,082.1), and the winning model in both the combined Iranian and combined British datasets was the EV + SD + R model. We finally combined all six datasets and again the tied winning models both included regret and risk (BIC EV + SD + R 6,913.3; EV + SD + D + R 6,914.3).

**Table 1:** Model comparison using summed BICs. Note. Winning models are underlined.

		EV + SD + R model	EV + SD + D + R model	EV + D + R model	EV + SD model	EV + D model	EV model
Iran	Exp1	1097.7	1112.1	1115.7	1128.3	1121.7	1200.5
	Exp2	1422.5	1427.9	1451	1438.9	1467.7	1507.2
	Combined	2474.8	2488.2	2520.4	2532	2556.5	2690.3
China	Exp1	847.7	855.3	840.9	890.7	850	913.6
	Exp2	1266.1	1257.3	1293.7	1314.3	1301.2	1375.5
	Combined	2080.7	2082.1	2104.1	2178.3	2139.3	2269.9
UK	Exp1	1067.2	1081.2	1076.8	1100.8	1092	1116.2
	Exp2	1405.2	1408.9	1417.2	1443.1	1432.8	1464.9
	Combined	2431.5	2443.6	2452.5	2515.4	2495.9	2563.4
All 6 datasets combined		6913.3	6914.3	7006.3	7174.2	7148.5	7493.3

We thus find that regret influences choice in all six datasets, and we find good evidence for the risk (SD) parameter. However, there was little evidence for the disappointment (D) parameter. The EV + D + R model lost to the EV + SD + R model in five of the six datasets, as well as when examining datasets combined across tasks for each of the three cultures and when combining all six datasets (Table 1). The more complicated EV + SD + D + R model does not consistently outperform the EV + SD + R model in any of the three cultures or when all six datasets are combined, while the EV + SD + R model outperforms it in five of the six datasets. Thus, we use the EV + SD + R model in our further analyses given that it performs consistently well across cultures, tasks and analyses.

Finally, we note that none of the six datasets showed a main effect of feedback condition (i.e. partial- or complete-feedback) or an interaction of regret or SD with feedback condition when these were added to the above analyses.

### Cultural impacts on risk in choice: Iranians are consistently risk seeking

**Figure 2:**
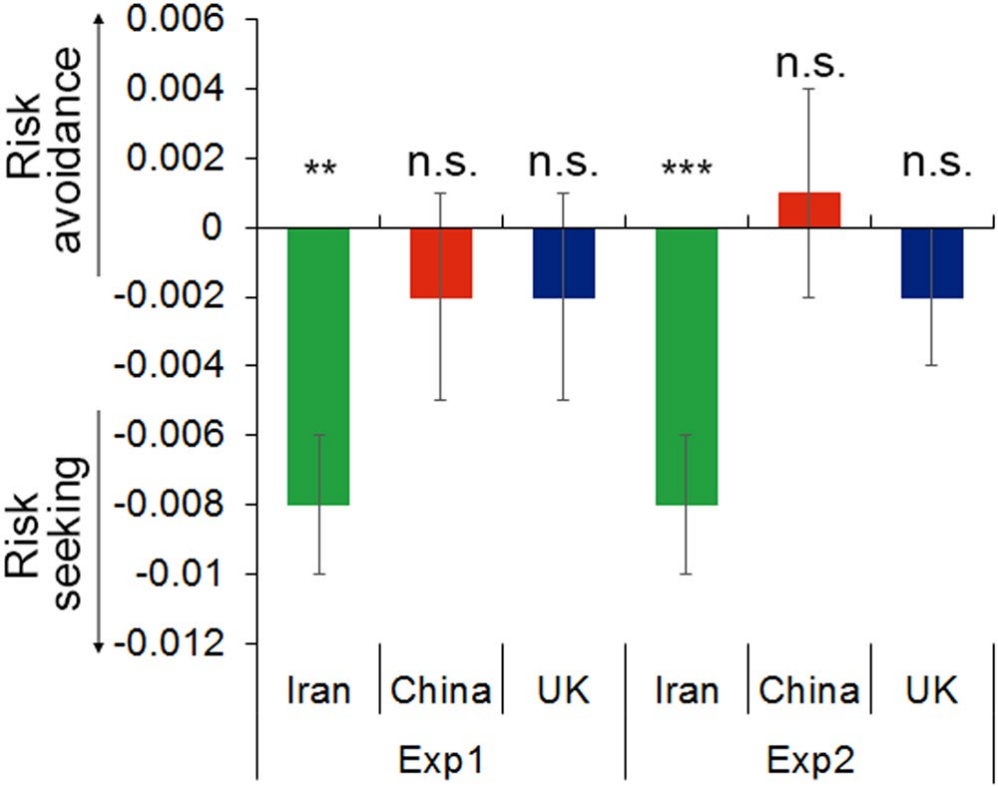
Choice behavior related to risk in each of the six datasets, analysed separately using the EV + SD + R model. Iranians are consistently risk-seeking across both task variants, while the UK and Chinese groups are risk-neutral in both task variants. Positive SD reflects risk-aversion, negative SD reflects risk-seeking. Error bars show standard error. *p < 0.05. **p < 0.01. ***p < 0.001.

Having shown that both risk and counterfactual thinking such as regret influences choice, we next examined these effects using our winning EV + SD + R model that quantifies risk-preference and regret-preference. First we consider risk, where positive SD coefficients reflect risk-avoidance, zero risk-neutrality and negative values risk-seeking. We examined each of the six datasets. Iranians were absolutely risk-seeking, and this was replicated across both task variants; for Exp1 (*n* = 19, SD = −0.008 ± standard error (s.e.m) 0.002, compared to risk-neutral z = −3.3, *p* = 0.001) and Exp2 (*n* = 20, SD = −0.008 ± 0.002, *z* = −4.4, *p* < 0.001) (Fig. 2). In contrast, the UK and Chinese groups were consistently risk-neutral, and again this was replicated across both task variants; for Exp1 in the UK (*n* = 19, SD = −0.002 ± 0.003, *z* = −0.82, *p* = 0.41), in China (*n* = 19, SD = −0.002 ± 0.003, *z* = −0.67, *p* = 0.51); and for Exp2 in the UK (*n* = 20, SD = −0.002 ± 0.002, *z* = −0.82, *p* = 0.41), in China (*n* = 20, SD = 0.001 ± 0.003, *z* = 0.42, *p* = 0.68) (Fig. 2, Supplementary Table S1).

We next combined all the datasets and derived model-based parameters for all 117 participants using the EV + SD + R model. Using the risk parameters in a 3 culture (Iran, China, UK) by 2 task variant (Exp1, Exp2) ANOVA revealed a main effect of culture (*F*(2, 111) = 8.2, *p* < 0.001, *η*^2^ = 0.13), no main effect of task variant (*F*(1, 111) = 0.6, *p* = 0.43, *η*^2^ = 0.005) and no interaction (*F*(2, 111) = 0.6, *p* = 0.53, *η*^2^ = 0.01). As expected from the above, this was driven by more risk taking in the Iranian datasets (SD = −0.0041 ± 0.001) compared to the UK datasets (SD = 0.0014 ± 0.001) (Tukey’s HSD: *p* = 0.0079), and the Chinese datasets (SD = 0.0027 ± 0.001) (Tukey’s HSD: *p* < 0.001). There was no difference between UK and Chinese datasets (Tukey’s HSD: *p* = 0.73).

### No culture shows a consistent direction for the impact of regret

Second, we considered regret. While regret influenced choice behaviour in all six datasets as shown above, no culture showed a consistent direction (i.e. regret seeking vs avoidance) across the two tasks for the impact of regret (as described below and see Supplementary Table S1) and there were no cultural differences. We used the regret parameters (from the EV + SD + R model across all 117 participants) in a 2 task × 3 culture ANOVA, which revealed no main effect of culture (*F*(2, 111) = 2.6, *p* = 0.08, *η*^2^ = 0.04), a main effect of task variant (*F*(1, 111) = 13.5, *p* < 0.001, *η*^2^ = 0.10) and no interaction (*F*(2, 111) = 2.0, *p* = 0.2, *η*^2^ = 0.03). Thus we saw no consistent effects of culture on anticipated regret. The effect of task dominated and led to greater regret avoidance in Exp1 than in Exp2 in all three cultures, although this was only significant for the Chinese datasets (R = 0.0025 ± 0.001 for Exp1 and R = −0.0026 ± 0.001 for Exp2; Tukey HSD *p* = 0.004), not in the UK (R = −0.0004 ± 0.001 for Exp1 and R = −0.0020 ± 0.001 for Exp2; *p* = 0.9) and Iran (R = 0.0020 ± 0.001 for Exp1 and R = −0.00003 ± 0.001 for Exp2; *p* = 0.7).

### Subjective ratings: Iranians show less response to negative outcomes

**Figure 3:**
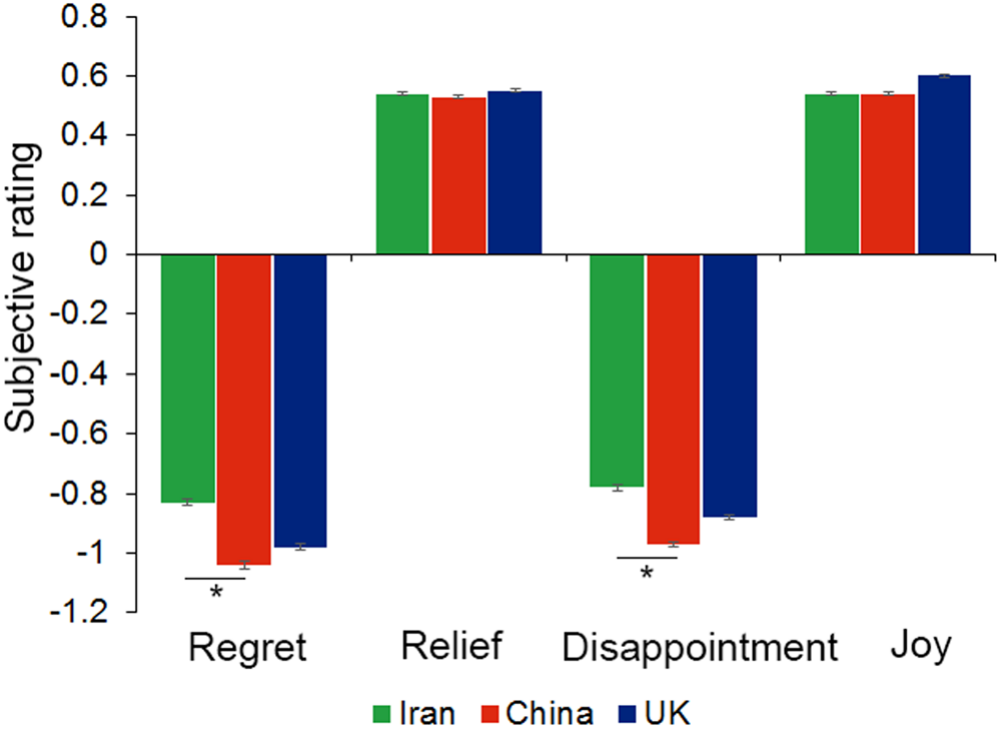
Subjective ratings. Iranians show the least subjective regret and disappointment. Error bars show standard error. *p < 0.05.

Next we asked if cultural impacts on subjective ratings in risky choice help explain differences in risky choice. After receiving feedback in each trial participants gave a subjective rating, and as in previous work we examined four types of trials^38^. We found selective cultural effects, with no cultural impact on responses to the two more positive types of outcomes (termed joy or relief), but cultural effects on responses to the two types of more negative outcomes (termed regret and disappointment) in both cases driven by a less negative Iranian response (Fig. 3).

In the complete feedback condition participants saw the outcomes of both lotteries and so may experience *regret* if the outcome of chosen option is worse than the outcome of unchosen option, or *relief* if the outcome of chosen option is better than the outcome of unchosen option. In the regret trials a 3 culture × 2 task variant ANOVA showed main effects of culture (*F*(2, 111) = 3.9, *p* = 0.023, *η*^2^ = 0.04) and task (*F*(1, 111) = 66.8, *p* < 0.001, *η*^2^ = 0.36), with no interaction (*F*(2, 111) = 0.57, *p* = 0.57, *η*^2^ = 0.01). Experienced regret was less in Iran (−0.83 ± 0.07) than China (−1.04 ± 0.07, Tukey HSD: *p* = 0.021), but there was no difference between Iranian and UK (−0.98 ± 0.06, Tukey HSD: *p* = 0.14) or Chinese and UK (Tukey HSD: *p* = 0.71) participants. Regret was less in Exp2 (−0.70 ± 0.04) than Exp1 (−1.21 ± 0.05). A similar ANOVA on the relief trials showed no main effects or interactions (*p* values > 0.08).

In the partial feedback condition participants only received feedback on the chosen lottery, and so may experience *disappointment* if that outcome is the worse outcome in the lottery or *joy* if it is the better outcome. For disappointment trials, a 3 culture × 2 task variant ANOVA again revealed main effects of culture (*F*(2, 111) = 4.3, *p* = 0.016, *η*^2^ = 0.06) and task (*F*(1, 111) = 19.5, *p* < 0.001, *η*^2^ = 0.14) with no interaction (*F*(2, 111) = 1.2, *p* = 0.31, *η*^2^ = 0.02). Experienced disappointment was less in Iran (−0.78 ± 0.06) than China (−0.97 ± 0.04) (Tukey HSD: *p* = 0.012), but there was no difference between Iranian and UK (−0.88 ± 0.04) (Tukey HSD: *p* = 0.26) or Chinese and UK (Tukey HSD: *p* = 0.37) participants. Disappointment was lower in Exp2 (−0.76 ± 0.04) than Exp1 (−0.99 ± 0.03). A similar ANOVA on the joy trials showed no main effects or interactions (*p* values > 0.36).

Finally, we note no difference to main effects of culture or interactions with culture reported above when using non-normalised ratings.

### Reaction times

We examined a 3 culture × 2 task variant ANOVA with each participant’s mean RT as the dependent variable. This revealed longer RTs in Exp2 than Exp1, as anticipated given the smaller EV differences between options within trials in Exp2 would likely increase their difficulty (*F*(1, 111) = 29.7, *p* < 0.001, η^2^ = 0.20). In keeping with our findings from choice and subjective ratings above, it also revealed a main effect of culture (*F*(2, 111) = 4.9, *p* = 0.009, *η*^2^ = 0.065), with no interaction (*F*(2, 111) = 0.08, *p* = 0.92, *η*^2^ = 0.0011) – and this was again driven the Iranian sample. There were longer RTs in Iran (mean = 2, 201 ± 60 ms; Exp1 = 2, 015 ± 70 ms; Exp2 = 2, 378 ± 81 ms) than in China (mean = 2, 000 ± 53 ms; Exp1 = 1, 845 ± 52 ms; Exp2 = 2, 147 ± 81 ms) (Tukey HSD: *p* = 0.023), or in UK (1, 995 ± 63 ms; Exp1 = 1, 822 ± 72 ms; Exp2 = 2, 159 ± 89 ms) (Tukey HSD: *p* = 0.019). Chinese and UK RTs did not differ (Tukey HSD: *p* = 0.998).

**Figure 4:**
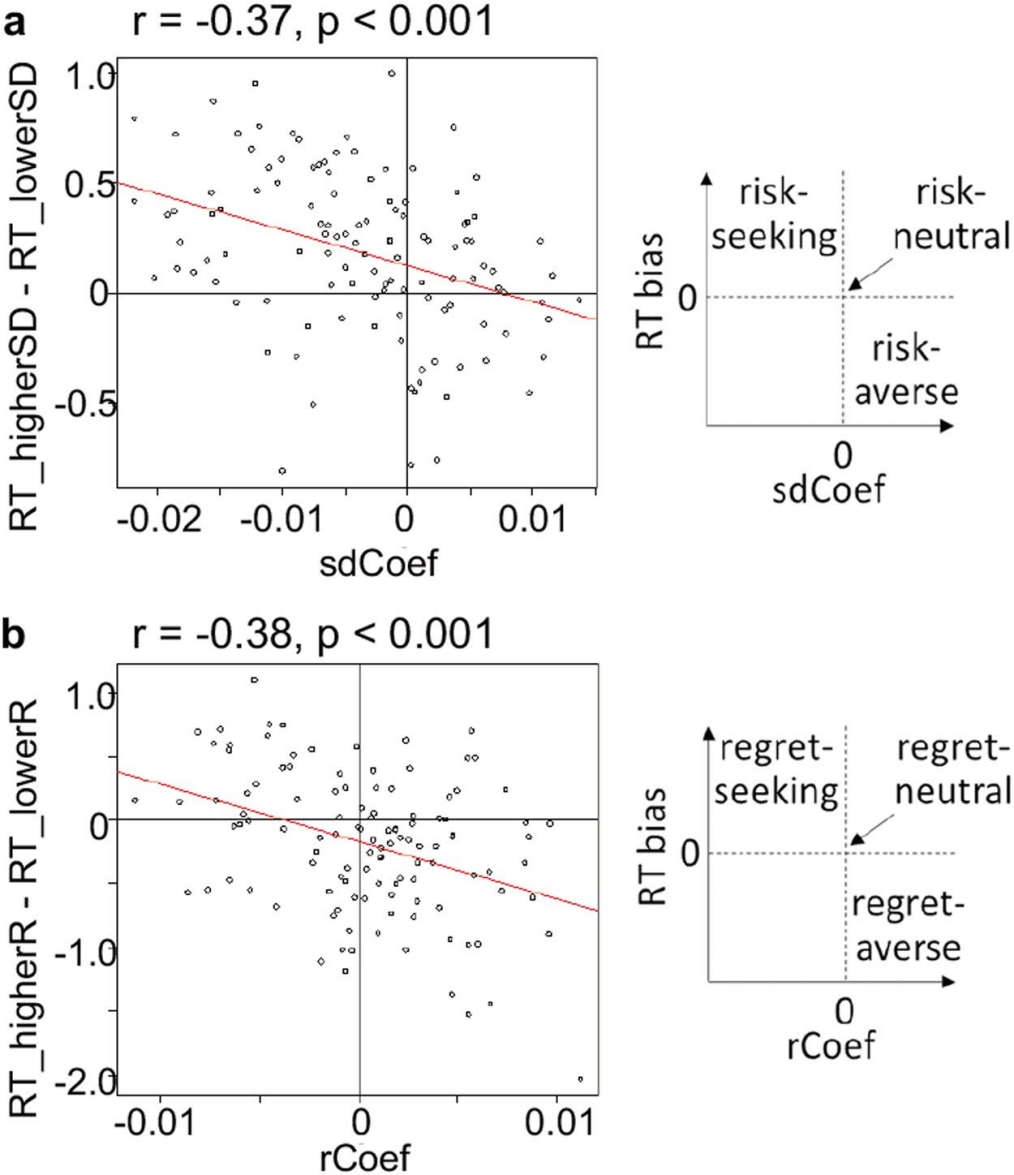
Reaction times. Both (**a**) risk and (**b**) regret biased reaction times as predicted by approach–avoidance mechanisms: As the effect of risk/regret depends on individuals’ subjective preference we looked between subjects. An individual’s risk/regret preference (i.e. the obtained coefficients of the EV + SD + R model of choice behaviour; denoted as sdCoef and rCoef, respectively) strongly predicted their RT bias (RThigherSD - RTlowerSD or RThigherR - RTlowerR). We observed our predicted pattern, where: risk slowed approach when risk was aversive; risk induced no RT bias when risk was neutral; and risk speeded approach when risk was appetitive (each panel on the right hand side is a cartoon of these predictions). Regression lines are shown, which are not constrained in any way. Grey lines show risk-neutrality in choice (i.e. sdCoef = 0 or rCoef = 0) and no RT bias (i.e. RThigherSD - RTlowerSD = 0 or RThigherR - RTlowerR = 0).

Reaction times (RTs) also showed influences of risk and anticipated regret (Fig. 4). These data were consistent with each acting as appetitive or aversive stimulus features, where it is known that individuals are slower to approach aversive stimuli and faster to approach appetitive stimuli^39^. We previously discussed such RT effects for risk^5,6,19^. Regarding risk, this can be aversive, neutral or appetitive depending on an individual’s risk preference (Fig. 4a). We found that individuals’ risk preference strongly predicted RT differences when approaching (choosing) the riskier relative to the less risky option (i.e. RThigherSD - RTlowerSD) (*r*(115) = −0.37, *p* < 0.001). Furthermore, the pattern was exactly as predicted where risk-averse individuals were slower to approach risk; risk neutral showed no RT difference; and risk-seeking subjects were faster to approach risk. These findings for risk showed consistency across culture and task (see Supplementary Results online). Regret showed the same pattern. Individuals’ regret preference strongly predicted the RT difference when approaching (choosing) the higher regret option with higher anticipated regret (*r*(115) = −0.38, *p* < 0.001). This was broadly consistent across culture and task (see Supplementary Results online).

### Gender did not alter effects of risk or regret on choice behaviour, subjective ratings or RTs

Finally, we note that gender was balanced in all six groups. Adding gender to the ANOVAs described showed no main effects or interactions of gender and the other results were unaltered.

## Discussion

We investigated the impact of culture on two key modulators of value-based choice—regret and risk—employing a probabilistic gambling task and quantitative analysis that implicitly assayed these components independently. We found robust commonalities across cultures, with our computational approach enabling us to identify that both regret and risk independently drove choice in all three cultures. Further, we identified cultural differences that, in this first cross-cultural controlled laboratory study of risk in Iran, centred on the Iranian compared to the Chinese and the UK participants. Iranians were the only culture consistently risk-seeking in choice, which was seen across both task variants. Moreover, regarding the process of choice this cultural effect accorded with two other findings: firstly the selectively reduced Iranian subjective response to the negative but not positive outcomes of risky choices, which provided an explanation for increased Iranian risk-taking; and secondly also with the RT differences seen only in the Iranian participants.

The pattern of cultural differences in risk-taking we observe, using precisely specified measures of risk across three diverse cultures, helps arbitrate between potential origins for cultural effects in value-based choice. Including Iran is important, as influential work has shown striking commonalities in value-based choice in a public goods game between China and the West, but distinct from behaviour in Middle Eastern cities culturally and geographically proximal to Iran, with such differences attributed to different norms and institutions^33^. This is in contrast to risk preference being determined by a greater social ‘cushion’^21^ facilitating risk-taking in more ‘collectivist’ societies (e.g. Iran and China^31^) versus the more individualist UK. Differences in norms and institutions between Iran compared to the UK and China include not only unpredictable exposure to war and military conscription (with such factors shown to affect value-based choice in other countries^34^) but also a Sharia financial system in which concepts such as gambling and interest-based investment are contested^35^. The similarity in UK and Chinese risk preference found here is also in keeping with the previously inconsistent findings across multiple studies of risk that used small numbers of trials and a broader operationalisations of risk^20–26^. As well as helping explain broader cross-cultural patterns, evidence on the specific Iranian case is also important, for instance where Iranian cultural attitudes to risk have been discussed in the context of public policy debates^40^.

Our results also speak to the existing mainly Western literature on regret and risk in value-based choice. Firstly, formal model comparison enabled us to show that in three diverse cultures a model including risk and regret better predicted choice than a previously used model instead including a ‘disappointment’ parameter^37^. Second, whilst risk-taking was strikingly consistent between task variants (Fig. 2), the impact of counterfactual thinking differed between the task variants. Given the larger EV differences between options in Exp1 providing greater potential for regret, as expected this was associated with greater regret avoidance, more negative subjective ratings reflecting experienced regret and faster RTs as more divergent EVs decreased choice difficulty. Further work can usefully examine these differences within cultures. Also, given the strong correlations of SD and regret differences in the Exp1 task, it is hard to appropriately parse the influence of each component on subject’s choice. In fact, in most of the existing literatures of quantitative counterfactual decision making studies, the influence of anticipated regret can always be re-interpreted by the potential contamination of gamble variance difference^37^. We thus conducted Exp2 task to further dissociate the individual contribution of SD and regret on individuals’ choice behaviour. Thirdly, although controlled for in our design, culture may be an important but underappreciated source of variance in data from highly diverse cities such as London. In London around 40% of the population were born outside the UK^41^, and at UCL^42^ around a third of students are from overseas of whom around half are from Asia and 5% from the Middle East.

Bringing together literature on risk and regret in the process of value-based choice also highlights areas for future work. Within the process of choice, one can consider a value modulator’s effects in at least three distinct time periods. Take regret as an example. In a first epoch the potential regret may be processed at the time of making the choice, called “anticipated regret” here and in e.g^37^. In a second epoch there may be regret-related processing after making the decision but before seeing the outcome. A third epoch involves regret-related processing at the time of seeing the outcome. In this study we examined regret-related processing in the first and third epochs – and future work may fruitfully examine the second epoch. We might expect distinct neural correlates of regret in this second epoch between decision and outcome. Indeed, work on examining risk-processing the first and second epochs (sometimes described as “decision-risk” and “anticipation-risk”^43^) has identified differences in risk encoding when comparing the first^43,44^ and second epochs^7^.

Finally, in light of the “replication crisis” we suggest that further work should seek to replicate these findings – and also test for additional potential sources of difference between these populations. Whilst we tested across task variants, balanced for gender and ensured our design was adequately powered, future work could for example systematically examine broader populations beyond the largely student groups examined here.

Computational approaches that can tightly specify and independently assay key modulators of value-based choice have enabled rapid advances in the primarily Western neurobiologically-grounded accounts of choice^8,9^ - and here enable us to show important commonalities and differences between cultures. Applying these in China, the West and Iran, our data provides convergent evidence for differences in a key 21st century culture—Iran—about which little was known previously, as well as new evidence to help arbitrate between causes of cultural differences between the West and China. Understanding value-based choice across cultures is increasingly important as it critically underpins the growing area of behavioural change across policy areas ranging from health to education.

## Methods

We tested the effects of risk and regret on financial decision-making in three distinct countries: China, Iran and the UK. Given the challenges of replicability we also tested two variants of the task, each in a new sample in each culture. We thus tested six groups of participants in a 3 culture × 2 task variant (‘Exp1’ and ‘Exp2’) design.

### Participants

A total of 117 healthy participants, with no history of mental or neurological disorders, took part after providing informed consent for the project which was approved by the locally relevant, authorised ethics committees. In the UK this was the UCL Research Ethics Committee; in Iran the Ethics Committee of the Faculty of Psychology and Educational Sciences, Tehran University; and in China the Ethics Committee of School of Psychological and Cognitive Sciences, Peking University. All methods were performed in accordance with the relevant guidelines and regulations. Supplementary Methods and Table S2 summarise age and gender for the six participant groups. Each group was balanced for gender and age did not differ between cultures.

The Chinese groups were tested at Peking University, the Iranians at Tehran University and the UK participants at University College London (UCL). London is highly ethnically diverse, and thus for the UK sample we only included participants who: (i) were born in the UK; (ii) had lived mainly in the UK in the first 10 years of their lives; and (iii) at least one parent was also born in the UK. All Chinese and Iranian participants were born, grew up and live in those countries. In all three countries recruitment was via standard means on campus such as flyers and mailing lists, and a majority of participants in all datasets were students.

### Experimental design

In current study, we adopted variants of a Wheel-of-Fortune task to measure participants’ risk and regret^36,37^. In each trial, participants were asked to select one of two lotteries. Each lottery had two potential outcomes, each indicated numerically. The probability of each outcome was shown by the size of the segment in the lottery. Each trial began with a blank screen presented for 1–2 s (mean = 1.5 s), followed by a 4 second choice period in which they viewed both options and input their choice by pressing a left or right button. After the 4 seconds of the choice period, the arrow span for 1 second and finished at a randomly-determined final location that indicated the outcome in the selected lottery. The outcome was displayed for 3 seconds, after which the participant rated their feeling about the outcome.

Our two task variants (Exp1 and Exp2) differed in two ways. First, the stimulus list differed. Exp1 used a stimulus list of 48 trials used previously by^37^, in which each lottery contained two outcomes (drawn from 200, 50, −50 and −200 points) with three levels of outcome probability (0.2, 0.5 and 0.8). The two lotteries in each trial always differed from one another. However, that existing stimulus list is potentially limited as most trials have a large difference in Expected Value (EV) between the two lotteries that may dominate subtle cultural effects of risk or anticipated regret. Further, there are high correlations between regressors of interest across the list (Tables 2 and 3). Thus the Exp2 stimulus list had smaller EV differences within pairs of lotteries (facilitated by using potential outcomes drawn from 200, 100, 50, −50, −100 and −200) and potential regressors of interest were less correlated across the 30 trials of the stimulus set. Supplementary Tables S3–S6 show the full stimulus lists and their descriptive statistics. Secondly, in light of previous work suggesting cultural differences in which initial trials in blocks affect Westerners more than Chinese^45^, we varied the ordering of feedback condition. In Exp1 the partial- and complete-feedback conditions were presented in blocks of 12 trials and before each block a screen told participants which feedback condition they were in. In Exp2 the trials were presented in a random mixed order throughout.

**Table 2:** Intercorrelations of Gamble Variables in stimulus set for Exp1, previously used by^37^. Note. *p < 0.05. **p < 0.01. ***p < 0.001.

	${\rm{\Delta }}$EV	${\rm{\Delta }}$SD	D
${\rm{\Delta }}$SD	−0.26		
D	0.30*	0.69***	
R	0.76***	−0.39**	−0.25

**Table 3:** Intercorrelations of Gamble Variables in stimulus set for Exp2. Note. *p < 0.05. **p < 0.01. ***p < 0.001.

	${\rm{\Delta }}$EV	${\rm{\Delta }}$SD	D
${\rm{\Delta }}$SD	0.33**		
D	0.55***	0.82***	
R	0.09	−0.23	−0.59***

All participants performed a practice session (12–15 trials). In the testing session they undertook the full stimulus list in random order presented once in each of the feedback conditions (i.e. 96 trials total in Exp1 and 60 total in Exp2). Participants were tested alone in the experimental room, with the experimenter waiting outside. Testing was conducted on computer using Psychtoolbox in Matlab. Total time for testing was approximately 18 mins for Exp1 and approximately 11 mins for Exp2. The mean number of missed trials was less than two in all six datasets, with no participant missing over 10 trials.

#### Language

Participants received the instructions on a written sheet in their local language (Mandarin in China, Farsi in Iran and English in the UK) that was also read out with them by the experimenter (see Supplementary Information online). These were first written in English and then translated into Chinese and Farsi, which was checked by local native speakers in the experimental team. The terms used for the axis of the subjective rating in the official languages were: in English “extremely positive,” “neither positive nor negative,” and “extremely negative”; in Chinese “” and “”; and in Farsi “,” and “”.

#### Payment

In all three countries participants were informed that their payment depended on their decisions in the experiment. This consisted of an endowment (China 25 yuan; UK 5GBP; Iran 120,000 Rial), plus the result of one trial picked at random and played out for real (with points converted to local currency at a rate in China of 0.1, equivalent to an amount between −20yuan and 20yuan; UK conversion rate 0.02, min. −4GBP, max 4GBP; Iran conversion rate 400, min. −80000rial, max 80000rial). The payment amounts are typical in the three institutions and are similar across the three countries relative to GDP per capita corrected for purchasing power parity^46^. Unknown to the participants in Iran and the UK, for local administrative reasons they did not receive amounts closer to the lower potential outcomes. All stimuli during the experiment were shown as points across the three cultures.

### Behavioural modelling of choice

Our model-based analysis of participants’ choices sought to determine whether our decision variables of interest—risk and regret—influence choice and to quantify them for further analysis. We estimated model parameters for participants using mixed-effects logistic regression models implemented with maximum likelihood estimators (MLEs) by using the glmer function in the R package lme4. We compared models with different utility functions, using the Bayesian information criterion (BIC).

Within different utility function models, for each trial we focus on the four gamble statistics (gamble expected value (EV), gamble variance (SD), gamble’s anticipated disappointment (D), and the anticipated regret (R)). These arise as each trial, which consists of left and right lotteries, has six pseudo-independent parameters: two possible outcomes of the left lottery, x_1_ and y_1_ (x_1_ > y_1_); the probability of earning x_1_, p (which entails that the probability of y_1_ is 1 − p); two possible outcomes of the right gamble, x_2_ and y_2_ (x_2_ > y_2_); and the probability of earning x_2_, q (the probability of y_2_ is 1 − q). We thus constructed different models based on the statistics described above and compared models’ performance, with each described below.

First, in a very simple EV Only model, individuals only cared about difference in the expected value (ΔEV) of the two options in a trial. ΔEV is the difference in expected value between the left and right gambles, namely:

1 $${\rm{\Delta }}EV=E{V}_{1}-E{V}_{2},$$

where EV is:

2 $$E{V}_{1}=p{x}_{1}+(1-p){y}_{1},\,E{V}_{2}=q{x}_{2}+(1-q){y}_{2},$$

Second, we asked whether choice was also influenced by risk, using a Mean–Risk model (EV + SD). Specifically, risk was measured as standard deviation of each gamble’s potential outcome. This model included both the ΔEV parameter and the ΔSD that was defined as the difference in weighted standard deviation, namely:

3 $${\rm{\Delta }}S{D}_{1}=S{D}_{2}-S{D}_{1},$$

where

4 $$S{D}_{1}=\sqrt{p{({x}_{1}-E{V}_{1})}^{2}+(1-p){({y}_{1}-E{V}_{1})}^{2}},\,S{D}_{2}=\sqrt{q{({x}_{2}-E{V}_{2})}^{2}+(1-q){({y}_{2}-E{V}_{2})}^{2}}.$$

Third, we asked whether both risk and regret influence choice, using a Mean-Risk-Regret model (EV + SD + R). R is a regret factor previously used by e.g.^37^. This anticipated regret is the absolute value of the difference between the lowest and the highest outcome across gambles, such that a participant can avoid future regret by choosing a gamble that minimizes this difference. To preempt our results, this was the winning model in five of our six datasets. R was defined as:

5 $$R=|{y}_{2}-{x}_{1}|-|{y}_{1}-{x}_{2}|.$$

The fourth and fifth models used a disappointment (D) parameter previously used by^37^ instead of ∆SD as in the above models. Disappointment arises when comparing outcomes on a selected gamble, in which the alternative outcome is more positive than an experienced outcome. The magnitude of disappointment reflects the discrepancy between the ‘unobtained outcome’ and actual outcome of the selected gamble. An individual can avoid anticipated disappointment by choosing a gamble that minimizes a difference between the lowest and highest outcome, weighted by the probability of the worst possible outcome^37^. This gave an EV + D model and an EV + D + R model. As in^37^, D was defined as:

6 $$D=|{y}_{2}-{x}_{2}|(1-q)-|{y}_{1}-{x}_{1}|(1-p).$$

The final EV + SD + D + R model included all four of the parameters above.

In all of the models, we modelled choice behaviour with a mixed-effects logistic regression model:

7 $${\rm{logit}}\,({\rm{\Pr }}\,({y}_{i,t}=1))={x}_{t}\beta +{z}_{t}{b}_{i}+\varepsilon ,$$

8 $${{\bf{b}}}_{i} \sim N(0,{\sigma }^{2}{\bf{S}}({\boldsymbol{\theta }}))$$

9 $$\varepsilon  \sim N(0,{\sigma }^{2}{\bf{I}})$$

where *y* is the choice (1: left option, 0: right option), *i* denotes subject number, *i* = 1, 2, … *N*, *t* denotes the trial number. *x*_*t*_ and *z*_*t*_ are the 1-by-*l* and 1-by-*m* row vectors, rows of design matrices corresponding to *t*-th trial of *i*-th subject; *l* and *m* are the number of fixed- and random-effect variables, respectively. *β* and b are the model parameters to be estimated. *β* is a *l* –by-1 fixed-effect vector, and b_*i*_ is a *m*-by-1 random-effect vector. *N* means a normal distribution. S is a symmetric and positive semidefinite matrix, parameterized by a variance component vector *θ*. We use a diagonal matrix for S assuming the independence of random-effect variables. I is an identity matrix, and *σ*^2^ is the error variance. The main assumption behind this model is that the coefficients of the fixed-effect variables *β* reflect the population level, while the random-effect variables *b* reflect the deviation from the population level for each subject. The random-effect coefficients *b*_*i*_ vary across subjects.

Models reported here do not include the factor of feedback condition (partial or complete) unless otherwise specified.

### Statistical analysis

In addition to the analyses above, other statistical tests used were z tests for logistic regression coefficients, analyses of variance (ANOVA) followed by post hoc Tukey Honest Significant Difference (HSD) tests, or Fisher z-transformation in R statistical computing software. Reported p-values are two-tailed.

### Subjective ratings

We standardized each individual’s subjective ratings by correcting the mean and dividing by the standard deviation, i.e. ipsatisation, to reduce cross-cultural response bias^47^.

### Reaction time analysis

We normalised each individual’s RTs by taking the natural logarithm, mean-correcting and dividing by the standard deviation. However, we found the same effects using “raw” or normalised RTs.

## Electronic supplementary material

Supplementary information

Supporting Information S1

Supporting Information S2

Supporting Information S3

Supporting Information S4

Supporting Information S5

Supporting Information S6

Supporting Information S7

Supporting Information S8

Supporting Information S9

## Data Availability

The datasets generated during and/or analysed during the current study are available from the corresponding author on reasonable request.
