# Supplementary material for: Parsing cultural impacts on regret and risk in Iran, China and the United Kingdom
Source: Sci Rep. 2018 Sep 14;8:13862. doi: 10.1038/s41598-018-30680-7 (PMC6138714; doi:10.1038/s41598-018-30680-7)
Supplement: Supplementary file 1 — Supplementary information [file 41598_2018_30680_MOESM1_ESM.doc]

# Supplementary information: Parsing cultural impacts on regret and risk in Iran, China and the United Kingdom

Li Li^†^, Shiro Kumano^†^, Anita Keshmirian, Bahador Bahrami^*^, Jian Li^*^ & Nicholas D. Wright^*^

* Corresponding Authors (E-mails: bbahrami@gmail.com; li.jian@pku.edu.cn; nick@nicholasdwright.com)

† Both authors contributed equally to the research

# Supplementary Methods

## Participants

For age, a 3 culture x 2 task variant ANOVA showed no main effect of culture (*F*(2,111) = 0.1, *p* = 0.89), no interaction (*F*(2,111) = 1.2, *p* = 0.30) although participants were slightly younger in the Exp1 task variant (mean = 22.9, ±standard deviation 3.0) than Exp2 (mean =24.6 ± 3.5; *F*(1,111) = 7.7, *p* = 0.007). Two further participants were excluded from Exp1, one in the UK who responded immediately on presentation of all stimuli and one in Iran who always chose the left option.

# Supplementary Results

## Reaction times showed influences of risk and anticipated regret.

Regarding risk, this can be aversive, neutral or appetitive depending on an individual’s risk preference (*Figure 4a*). We found that individuals’ risk preference strongly predicted RT differences when approaching (choosing) the riskier relative to the less risky option (i.e. RThigherSD - RTlowerSD) (*r*(115) = -0.37, *p* < 0.001). Furthermore, the pattern was exactly as predicted where risk-averse individuals were slower to approach risk; risk neutral showed no RT difference; and risk-seeking subjects were faster to approach risk.

These findings for risk showed consistency across culture and task. We observed the same relationship when separately examining data for Iran (*r*(37) = -0.49, *p* = 0.0014) and China (*r*(37) = -0.36, *p* = 0.023), although not the UK (*r*(37) = -0.13, *p* = 0.42). Culture did not affect these relationships when comparing the *r* coefficients using Fisher z-transformation (China and Iran, *z* = 0.69, *p* = 0.49; China and UK, *z* = -1.05, *p* = 0.29; Iran and UK, *z* = -1.74, *p* = 0.082). Correlations were significant for both Exp1 (*r*(55) = -0.41, *p* = 0.0016) and Exp2 (*r*(58) = -0.37, *p* = 0.0033) and *r* values did not differ between these tasks (*z* = -0.22, *p* = 0.83).

Regret showed the same pattern. Individuals’ regret preference strongly predicted the RT difference when approaching (choosing) the higher regret option with higher anticipated regret (*r*(115) = -.38, *p* < 0.001). This was broadly consistent across culture and task. We observed the same relationship when separately examining data for Iran (*r*(37) = -0.54, *p* < 0.001) and China (*r*(37) = -0.34, *p* = 0.035), although not the UK (*r*(37) = -0.30, *p* = 0.06). Culture did not affect the *r* values (Fisher z-transformation: China and Iran, *z* = 1.06, *p* = 0.29; China and UK, *z* = -0.16, *p* = 0.87; Iran and UK, *z* = -1.22; *p* = 0.22). The correlation was significant for Exp2 (*r*(58) = -0.29, *p* = 0.023) although not for Exp1 (*r*(55) = -0.23, *p* = 0.080), and these did not differ between tasks (*z* = 0.33, *p* = 0.74).

# Supplementary Tables

**Table S1** Fixed Effects for Group (and Standard Error) of EV+SD+R Model

|  | Exp1 | | |  | Exp2 | | |
| --- | --- | --- | --- | --- | --- | --- | --- |
| Variables | Iran | China | UK |  | Iran | China | UK |
| Intercept | 0.294*  (0.113) | 0.226  (0.12) | 0.196  (0.102) |  | 0.207**  (0.069) | -0.111  (0.078) | -0.127  (0.087) |
| ΔEV | 0.024***  (0.002) | 0.031***  (0.003) | 0.028***  (0.004) |  | 0.023***  (0.002) | 0.032***  (0.003) | 0.024***  (0.002) |
| ΔSD | -0.008**  (0.002) | -0.002  (0.003) | -0.002  (0.003) |  | -0.008***  (0.002) | 0.001  (0.003) | -0.002  (0.002) |
| R | 0.003**  (0.001) | 0.004**  (0.001) | 0.0002  (0.001) |  | 0.001  (0.001) | -0.003*  (0.001) | -0.002  (0.001) |

*Note.* Each of the six datasets are analysed on their own.

* *p* < .05. ** *p* < .01. *** *p* < .001

**Table S2** Demographic Details

|  |  | Age | | |  |
| --- | --- | --- | --- | --- | --- |
| Dataset | n | M | SD | Range | Gender (m:f) |
| Exp1 |  |  |  |  |  |
| Iran | 19  (+1 excl) | 22.2 | 2.5 | 19-28 | 9:10 |
| China | 19 | 23.1 | 2.6 | 20-27 | 10:9 |
| UK | 19  (+1 excl) | 23.6 | 3.7 | 18-30 | 10:9 |
| Exp2 |  |  |  |  |  |
| Iran | 20 | 25.0 | 4.4 | 19-33 | 10:10 |
| China | 20 | 24.8 | 2.8 | 21-29 | 9:11 |
| UK | 20 | 24.1 | 3.3 | 19-31 | 11:9 |

**Table S3** Gamble List and Summary Statistics for Exp1 (Taken from(Coricelli et al., 2005))

| No. | x1 | p | y1 | 1-p | x2 | q | y2 | 1-q | ΔEV | ΔSD | D | R |
| --- | --- | --- | --- | --- | --- | --- | --- | --- | --- | --- | --- | --- |
| 1 | 200 | 0.8 | 50 | 0.2 | 200 | 0.2 | 50 | 0.8 | 90 | 0 | 90 | 0 |
| 2 | 200 | 0.2 | 50 | 0.8 | 200 | 0.5 | 50 | 0.5 | -45 | 15 | -45 | 0 |
| 3 | 200 | 0.5 | -50 | 0.5 | 50 | 0.5 | -50 | 0.5 | 75 | -75 | -75 | 150 |
| 4 | 50 | 0.8 | -50 | 0.2 | 200 | 0.2 | -200 | 0.8 | 150 | 120 | 300 | 0 |
| 5 | 50 | 0.2 | -50 | 0.8 | 200 | 0.5 | 50 | 0.5 | -155 | 35 | -5 | -250 |
| 6 | 200 | 0.5 | -200 | 0.5 | 200 | 0.8 | 50 | 0.2 | -170 | -140 | -170 | -250 |
| 7 | 50 | 0.8 | -200 | 0.2 | 200 | 0.5 | -50 | 0.5 | -75 | 25 | 75 | -300 |
| 8 | 200 | 0.5 | 50 | 0.5 | 50 | 0.5 | -50 | 0.5 | 125 | -25 | -25 | 250 |
| 9 | 50 | 0.8 | -200 | 0.2 | 200 | 0.8 | -200 | 0.2 | -120 | 60 | 30 | -150 |
| 10 | 50 | 0.2 | -50 | 0.8 | -50 | 0.5 | -200 | 0.5 | 95 | 35 | -5 | 250 |
| 11 | 50 | 0.5 | -50 | 0.5 | 50 | 0.5 | -200 | 0.5 | 75 | 75 | 75 | 150 |
| 12 | 50 | 0.2 | -200 | 0.8 | 50 | 0.2 | -50 | 0.8 | -120 | -60 | -120 | -150 |
| 13 | 200 | 0.5 | -200 | 0.5 | 50 | 0.5 | -200 | 0.5 | 75 | -75 | -75 | 150 |
| 14 | 50 | 0.2 | -200 | 0.8 | 50 | 0.8 | -200 | 0.2 | -150 | 0 | -150 | 0 |
| 15 | 200 | 0.8 | -200 | 0.2 | 50 | 0.2 | -200 | 0.8 | 270 | -60 | 120 | 150 |
| 16 | 50 | 0.5 | -200 | 0.5 | 50 | 0.8 | -200 | 0.2 | -75 | -25 | -75 | 0 |
| 17 | 200 | 0.5 | -200 | 0.5 | 50 | 0.8 | -50 | 0.2 | -30 | -160 | -180 | 0 |
| 18 | 200 | 0.8 | -50 | 0.2 | 200 | 0.5 | 50 | 0.5 | 25 | -25 | 25 | -100 |
| 19 | -50 | 0.2 | -200 | 0.8 | 200 | 0.2 | -200 | 0.8 | -50 | 100 | 200 | -250 |
| 20 | -50 | 0.2 | -200 | 0.8 | 200 | 0.5 | -200 | 0.5 | -170 | 140 | 80 | -250 |
| 21 | 200 | 0.8 | 50 | 0.2 | 200 | 0.5 | -50 | 0.5 | 95 | 65 | 95 | 100 |
| 22 | 200 | 0.2 | -200 | 0.8 | 50 | 0.2 | -50 | 0.8 | -90 | -120 | -240 | 0 |
| 23 | 200 | 0.8 | -50 | 0.2 | 50 | 0.8 | -50 | 0.2 | 120 | -60 | -30 | 150 |
| 24 | -50 | 0.8 | -200 | 0.2 | 200 | 0.2 | -200 | 0.8 | 40 | 100 | 290 | -250 |
| 25 | -50 | 0.5 | -200 | 0.5 | 200 | 0.8 | -200 | 0.2 | -245 | 85 | 5 | -250 |
| 26 | -50 | 0.8 | -200 | 0.2 | 200 | 0.8 | -50 | 0.2 | -230 | 40 | 20 | -400 |
| 27 | -50 | 0.2 | -200 | 0.8 | 50 | 0.8 | -200 | 0.2 | -170 | 40 | -70 | -100 |
| 28 | -50 | 0.8 | -200 | 0.2 | -50 | 0.5 | -200 | 0.5 | 45 | 15 | 45 | 0 |
| 29 | 50 | 0.5 | -50 | 0.5 | 50 | 0.2 | -200 | 0.8 | 150 | 50 | 150 | 150 |
| 30 | 200 | 0.8 | -200 | 0.2 | -50 | 0.2 | -200 | 0.8 | 290 | -100 | 40 | 250 |
| 31 | 200 | 0.5 | -50 | 0.5 | -50 | 0.5 | -200 | 0.5 | 200 | -50 | -50 | 400 |
| 32 | -50 | 0.8 | -200 | 0.2 | 200 | 0.2 | -50 | 0.8 | -80 | 40 | 170 | -400 |
| 33 | 200 | 0.2 | -50 | 0.8 | 200 | 0.8 | 50 | 0.2 | -170 | -40 | -170 | -100 |
| 34 | 200 | 0.8 | -50 | 0.2 | 50 | 0.8 | -50 | 0.2 | 120 | -60 | -30 | 150 |
| 35 | 50 | 0.2 | -50 | 0.8 | 200 | 0.5 | 50 | 0.5 | -155 | 35 | -5 | -250 |
| 36 | 200 | 0.5 | 50 | 0.5 | 50 | 0.2 | -50 | 0.8 | 155 | -35 | 5 | 250 |
| 37 | 200 | 0.5 | 50 | 0.5 | 200 | 0.8 | -50 | 0.2 | -25 | 25 | -25 | 100 |
| 38 | 50 | 0.8 | -50 | 0.2 | 200 | 0.8 | -50 | 0.2 | -120 | 60 | 30 | -150 |
| 39 | 50 | 0.5 | -50 | 0.5 | 50 | 0.5 | -200 | 0.5 | 75 | 75 | 75 | 150 |
| 40 | 200 | 0.8 | 50 | 0.2 | 200 | 0.5 | -50 | 0.5 | 95 | 65 | 95 | 100 |
| 41 | 200 | 0.5 | -50 | 0.5 | 50 | 0.8 | -200 | 0.2 | 75 | -25 | -75 | 300 |
| 42 | 50 | 0.5 | -200 | 0.5 | 200 | 0.5 | -200 | 0.5 | -75 | 75 | 75 | -150 |
| 43 | 50 | 0.8 | -200 | 0.2 | 200 | 0.8 | -200 | 0.2 | -120 | 60 | 30 | -150 |
| 44 | 200 | 0.5 | -200 | 0.5 | -50 | 0.2 | -200 | 0.8 | 170 | -140 | -80 | 250 |

Table S3 (continued)

| No. | x1 | p | y1 | 1-p | x2 | q | y2 | 1-q | ΔEV | ΔSD | D | R |
| --- | --- | --- | --- | --- | --- | --- | --- | --- | --- | --- | --- | --- |
| 45 | 200 | 0.2 | -200 | 0.8 | -50 | 0.8 | -200 | 0.2 | -40 | -100 | -290 | 250 |
| 46 | 200 | 0.8 | -200 | 0.2 | 200 | 0.5 | -50 | 0.5 | 45 | -35 | 45 | -150 |
| 47 | 200 | 0.5 | -50 | 0.5 | 200 | 0.8 | -200 | 0.2 | -45 | 35 | -45 | 150 |
| 48 | 200 | 0.5 | -200 | 0.5 | 200 | 0.2 | -200 | 0.8 | 120 | -40 | 120 | 0 |

**Table S4** Summary Statistics for Exp1

| Variable | Min | Max | Mean | SD | Mean abs. |
| --- | --- | --- | --- | --- | --- |
| ΔEV | -245 | 290 | 1 | 132 | 114 |
| ΔSD | -160 | 140 | 0.4 | 72 | 60 |
| D | -290 | 300 | 5.2 | 119 | 90 |
| R | -400 | 400 | -4.2 | 199 | 164 |

*Note.* Mean Abs. = Mean absolute i.e. Mean (|x|).

**Table S5** Gamble List and Summary Statistics for Exp2

| No. | x1 | p | y1 | 1-p | x2 | q | y2 | 1-q | ΔEV | ΔSD | D | R |
| --- | --- | --- | --- | --- | --- | --- | --- | --- | --- | --- | --- | --- |
| 1 | 100 | 0.2 | -50 | 0.8 | 50 | 0.8 | -200 | 0.2 | -20 | 40 | -70 | 200 |
| 2 | 200 | 0.2 | -50 | 0.8 | 100 | 0.8 | 50 | 0.2 | -90 | -80 | -190 | 0 |
| 3 | 200 | 0.2 | -200 | 0.8 | -50 | 0.8 | -200 | 0.2 | -40 | -100 | -290 | 250 |
| 4 | 50 | 0.5 | -200 | 0.5 | 50 | 0.2 | -50 | 0.8 | -45 | -85 | -45 | -150 |
| 5 | 50 | 0.8 | -50 | 0.2 | 200 | 0.8 | -100 | 0.2 | -110 | 80 | 40 | -100 |
| 6 | 200 | 0.5 | -200 | 0.5 | 50 | 0.8 | -50 | 0.2 | -30 | -160 | -180 | 0 |
| 7 | 200 | 0.5 | -50 | 0.5 | 100 | 0.8 | 50 | 0.2 | -15 | -105 | -115 | 0 |
| 8 | 50 | 0.2 | -50 | 0.8 | 50 | 0.5 | -200 | 0.5 | 45 | 85 | 45 | 150 |
| 9 | 200 | 0.8 | -50 | 0.2 | 200 | 0.5 | 50 | 0.5 | 25 | -25 | 25 | -100 |
| 10 | 50 | 0.8 | -50 | 0.2 | 200 | 0.5 | -50 | 0.5 | -45 | 85 | 105 | -150 |
| 11 | 100 | 0.8 | 50 | 0.2 | 200 | 0.2 | 50 | 0.8 | 10 | 40 | 110 | -100 |
| 12 | 100 | 0.5 | -200 | 0.5 | 50 | 0.8 | -200 | 0.2 | -50 | -50 | -100 | 50 |
| 13 | 200 | 0.2 | -100 | 0.8 | 50 | 0.8 | -200 | 0.2 | -40 | -20 | -190 | 250 |
| 14 | 200 | 0.5 | -200 | 0.5 | 50 | 0.8 | -200 | 0.2 | 0 | -100 | -150 | 150 |
| 15 | 200 | 0.8 | 50 | 0.2 | 200 | 0.5 | 100 | 0.5 | 20 | -10 | 20 | -50 |
| 16 | 100 | 0.8 | -200 | 0.2 | 200 | 0.5 | -50 | 0.5 | -35 | 5 | 65 | -250 |
| 17 | 200 | 0.5 | -50 | 0.5 | 50 | 0.8 | -50 | 0.2 | 45 | -85 | -105 | 150 |
| 18 | -100 | 0.8 | -200 | 0.2 | -50 | 0.5 | -200 | 0.5 | 5 | 35 | 55 | -50 |
| 19 | 50 | 0.5 | -50 | 0.5 | 100 | 0.5 | -200 | 0.5 | 50 | 100 | 100 | 100 |
| 20 | 50 | 0.2 | -50 | 0.8 | 200 | 0.2 | -100 | 0.8 | 10 | 80 | 160 | -100 |
| 21 | 50 | 0.8 | -200 | 0.2 | 200 | 0.5 | -200 | 0.5 | 0 | 100 | 150 | -150 |
| 22 | 200 | 0.5 | -200 | 0.5 | 200 | 0.2 | -50 | 0.8 | 0 | -100 | 0 | -150 |
| 23 | 100 | 0.5 | -50 | 0.5 | 50 | 0.8 | -50 | 0.2 | -5 | -35 | -55 | 50 |
| 24 | 50 | 0.8 | -50 | 0.2 | 200 | 0.2 | -50 | 0.8 | 30 | 60 | 180 | -150 |
| 25 | 200 | 0.2 | -100 | 0.8 | 50 | 0.8 | -50 | 0.2 | -70 | -80 | -220 | 100 |
| 26 | 200 | 0.5 | 50 | 0.5 | 100 | 0.8 | 50 | 0.2 | 35 | -55 | -65 | 100 |
| 27 | 100 | 0.5 | -50 | 0.5 | 200 | 0.2 | -50 | 0.8 | 25 | 25 | 125 | -100 |
| 28 | 200 | 0.2 | -200 | 0.8 | 50 | 0.2 | -50 | 0.8 | -90 | -120 | -240 | 0 |
| 29 | -50 | 0.5 | -100 | 0.5 | -50 | 0.8 | -200 | 0.2 | 5 | 35 | 5 | 100 |
| 30 | 50 | 0.5 | -100 | 0.5 | 100 | 0.2 | -200 | 0.8 | 115 | 45 | 165 | 50 |

**Table S6** Summary Statistics for Exp2

| Variable | Min | Max | Mean | SD | Mean abs. |
| --- | --- | --- | --- | --- | --- |
| ΔEV | -110 | 115 | -8.8 | 48 | 37 |
| ΔSD | -160 | 100 | -13 | 76 | 68 |
| D | -290 | 180 | -22 | 132 | 112 |
| R | -250 | 250 | 3.3 | 131 | 110 |

*Note.* Mean Abs. = Mean absolute i.e. Mean (|x|).

**Instructions in each of the three countries**

**Iranian instruction sheet for Exp1**

**دستورالعمل**

به شرکت در آزمایش ما خوش آمدید و از شما متشکریم. این آزمایش درباره تصمیم گیری اقتصادی است. هدف از انجام این آزمایش این است که تا حد امکان امتیاز گرفته و تا آنجا که ممکن است تلاش کنید امتیازی از دست ندهید. در هر راند، از شما خواسته می شود که بین دو گزینه تصمیم بگیرید. آزمایش مطابق زیر است :

**جلسه تصمیم گیری فردی**

در موقعیت تصمیم‌گیری فردی از شما خواسته می شود که به تنهایی تصمیم بگیرید. در هر راند دو دایره نشان دهنده دو شرط بندی متفاوت است که در دو قسمت مخالف روی صفحه نشان داده می شود. دو نتیجه احتمالی هر شرط بندی روی قسمت چپ و راست دایره با عدد نشان داده می شود. اندازه نسبی رنگ آبی و قرمز روی هر قطاع دایره نشان دهنده احتمال نتیجه ممکنی است که می توانید کسب کنید. (مثال زیر را مشاهده کنید)

مثال


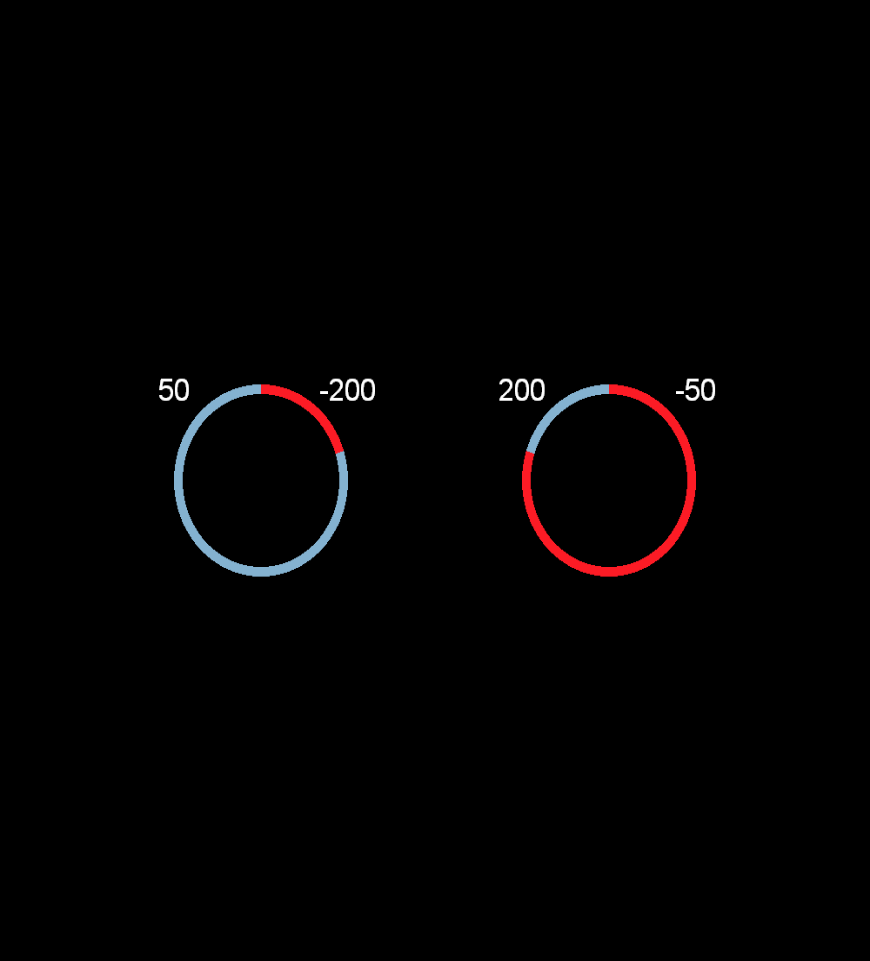


شرط بندی چپ: 80 درصد به دست آوردن 50 امتیاز، 20 درصد از دست دادن 200 امتیاز

شرطبندی راست: 20 درصد به دست آوردن 200 امتیاز، 80 درصد از دست دادن 50 امتیاز

لطفا برای انتخاب بین شرط بندی چپ و راست کلید های f و j را فشار دهید. بعد از این که تصمیم گرفتید، شرطبندی منتخب شما با یک مربع سبز مشخص خواهد شد. سپس پیکانی در مرکز دایره‌ی انتخاب شده پدیدار شده و شروع به چرخیدن میکند. مکانی که پیکان متوقف شود نتیجه آن راند را مشخص می کند. در برخی از راندها نتیجه‌ی حاصل از شرطبندی که شما انتخاب نکردید نیز نشان داده می شود. سپس از شما خواسته میشود که حس خود را نسبت به نتیجه حاصل شده روی محوری بین 50- تا 50 مشخص کنید(بسیار منفی تا بسیار مثبت). شما باید با استفاده از کلید f یا j نشانگر را به سمت مثبت یا منفی هدایت کنید. وقتی که نشان گر روی عدد مورد نظر شما قرار گرفت لطفا با فشردن کلید space آن را تایید کنید. در تمامی راندها شما باید در 4 ثانیه تصمیم‌گیری کنید. در غیر این صورت بدترین نتیجه هر راند را خواهید گرفت.

در ابتدای آزمایش به شما امتیازی اعطا خواهد شد که معدال۱۲۰,۰۰۰ ریال است. در انتهای آزمایش، یکی از راندها به شکل تصادفی انتخاب خواهد شد و نتیجه آن به عنوان دریافتی آزمایش با ضریب ۴۰۰ تبدیل خواهد شد. در نهایت میزان امتیاز شما شامل میزان اعطایی اولیه، و دریافتی از آزمایش است. در ادامه به چند سوال درباره آزمایش پاسخ داده و 30 راند برای آشنایی با دستورالعمل‌ها تمرین کنید تا مطمئن شوید که با روند آزمایش آشنا شده اید.

**Chinese instruction sheet for Exp1**

指导语

欢迎参加我们的实验!

这是一个关于经济决策的实验。在每一轮中，你将会被要求在两个赌博选项中选择其中一个。这个实验的目的是尽可能多地赢钱或者尽可能少地输钱。

实验流程如下：

这个实验以小组块的形式进行。有两种类型的组块，“部分反馈”组块和“全部反馈”组块，它们的呈现顺序被随机排列。在每一个组块开始的时候，屏幕上会告诉你现在是哪种组块。实验过程中请保持注意力集中。

在每一个组块内，每一轮开始之后，两个不同的赌博选项会以圆圈的形式呈现在屏幕的左右两边。每个赌博可能出现的两种结果会以数字的形式分别呈现在它左右两边。圆圈上蓝色和红色弧形的相对大小表示你可能得到左右两种结果的概率(看以下例子)。

例子：


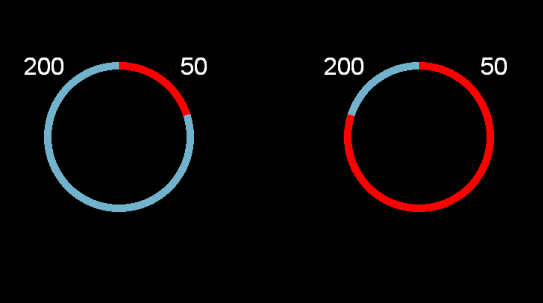


左边：80% 获得200点，20% 获得50点；

右边：20% 获得 200 点，80% 获得50 点

你可以通过按键盘上的“f”键或者“j”键来选择左边或者右边的选项。 一旦你做出选择，被选的选项就会被一个绿色的正方形框住。之后，一个箭头会出现在圆圈的中央并开始转动。箭头停止的位置表示这一轮你的所得。在“部分反馈”组块中，只有被选的选项的结果会呈现给你。在“全部反馈”组块中，被选的和没有被选的选项结果都会呈现给你。之后，你会被要求在一条-50（非常消极）到50（非常积极）的数轴上标出你对于这个结果的感受。你可以通过按 “ f ” 或 “ j ” 键来调节光标向更消极或者向更积极方向移动。当光标移动到你想要的位置时，请按下空格键进行确认。

实验过程中请保持注意力集中。每一轮，你必须在4s内做出选择，不然，你将得到那一轮中最坏的那个结果。实验开始前，你会得到20元本金。实验结束后，会随机抽取一轮，并以0.1的换算率把这一轮的结果转换成元，作为你此次实验的收益。最后，你得到的报酬包括基本被试费，本金和实验的收益。

实验开始之前，你需要回答一些问题和做12轮练习，目的是确保你理解我们的指导语和熟悉我们的流程。

问卷

以下问题是为了检查你是否正确理解以上的指导语。

1 如果你选择下图左边选项，你有更大的可能得到50元，这句话对吗? 如果选择右边的选项呢？


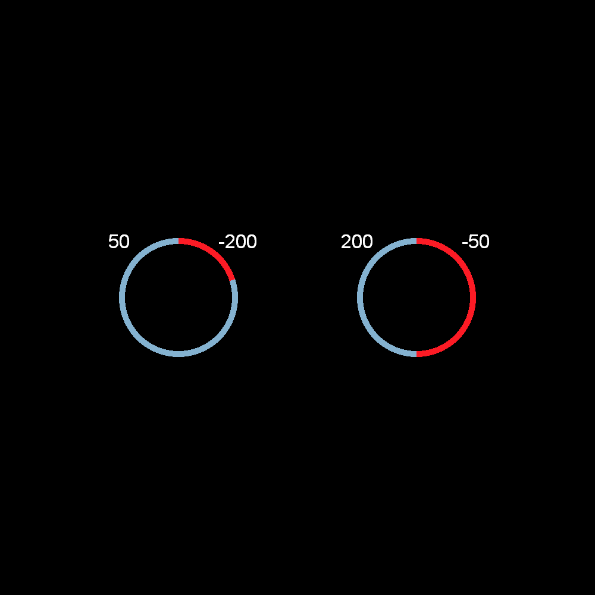


2 如果你想要选择左边的选项，要按哪个键？

3 如果你没有在4s中做出选择，结果会是什么？

4 下面情境，你将会得到/失去多少钱？


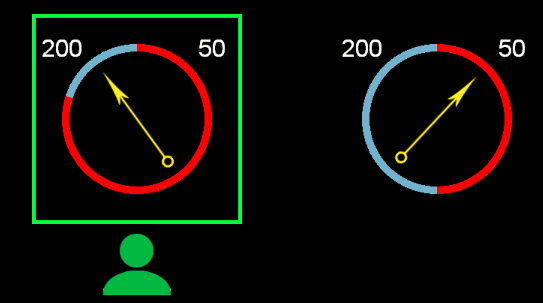

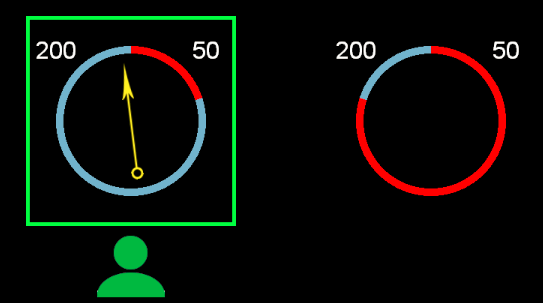


**British instruction sheet for Exp1**

Instructions

Welcome and thank you for participating in our experiment. This experiment is about the financial decision making. In each round, you will be asked to make a choice between two gambles. The goal of the experiment is to gain as much as possible or to lose as less as possible.

The experimental procedure is as follows:

This experiment is proceeded mini block by mini block. Two kinds of blocks, **Partial feedback block** and **Complete feedback block**, are randomly arranged. At the beginning of each block, the screen will tell you what kind of block the current one is. Please always keep attention during the experiment.

For each round within each block, two circles representing two different gambles will appear on contrary side of the screen. Two possible outcomes of each gamble will be displayed on its left and right side indicated by numbers. The relative size of blue and red sectors of the circle indicate the probability of the left and right outcomes you could receive (see the example below).

Example


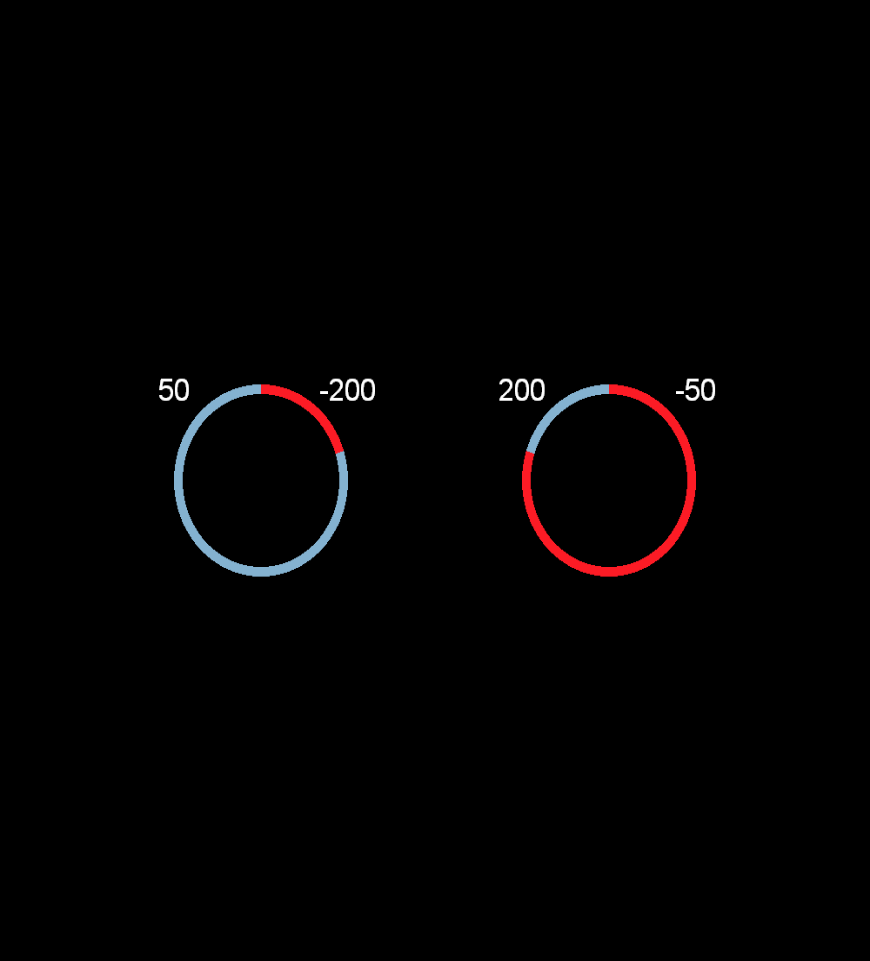


Left gamble：80% gain 50 point，20% lose 200 point；

Right gamble：20% gain 200 point，80% lose 50 point.

P

Please **press the button of “f” or “j”** on the keyboard **to choose the left or right gamble**. After you make a decision, the selected gamble will be highlighted with a green square. After this, an arrow will appear in the centre of the selected circle and begin spinning. The place where the arrow rests indicates your outcome in this round. In the **Partial feedback block**, only the outcome of the chosen gamble will be shown for you. In the **Complete feedback block**, the outcome of chosen and unchosen gamble will both be shown for you. And then you will be asked to rate your feelings about that outcome on a number axis of the range from -50 (extremely negative feeling) to 50 (extremely positive feeling). You should **press “ f ” or “ j ”** button to adjust the cursor toward negative or positive side. When the cursor reaches your target number, please **confirm it by pressing the space key**.

In all rounds, you should make your decision in 4 secs. If not, you will receive the worst outcome in that round. At the beginning, you will receive an endowment of £4. At the end of the experiment, one round will be randomly selected and its outcome as the experimental earning will be converted to £ at a rate of 0.02. Finally, your earning will include the basic payoff, the endowment and experimental earning.

Before proceeding the experiment, you should answer some questions and practice 12 rounds to make sure you understand the instructions and are familiar with the procedure.

Questionnaire

The goal of this part is to check whether you understand the instructions above correctly.


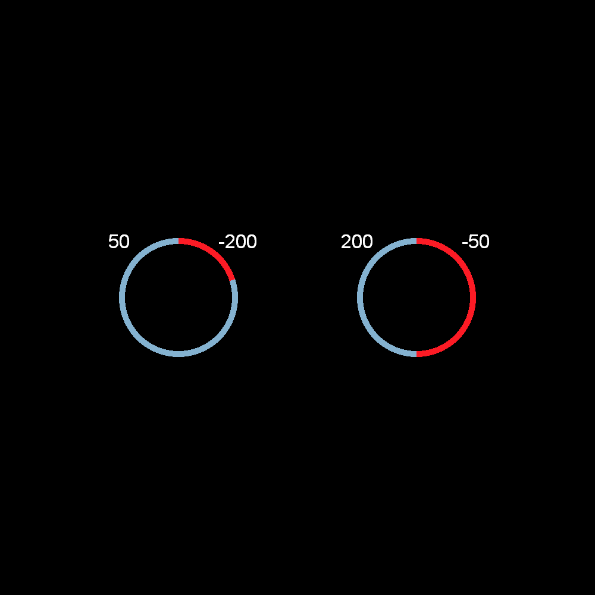
1 Are you more likely to gain 50 points than to lose 200 points if you choose the left gamble in the figure below? What about choosing the right one?

2 If you want to choose the left option, which button should you press?

3 If you do not make decision in 4 secs, what will happen?

4 How many points can you receive in the following cases?

1. **Partial feedback block** (2) **Complete feedback block**


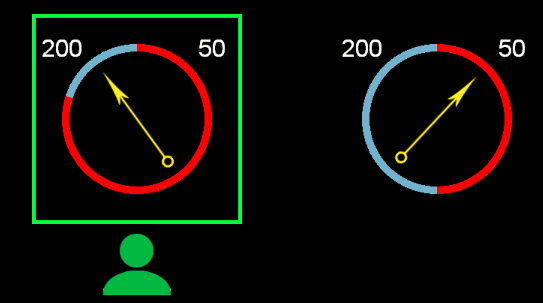

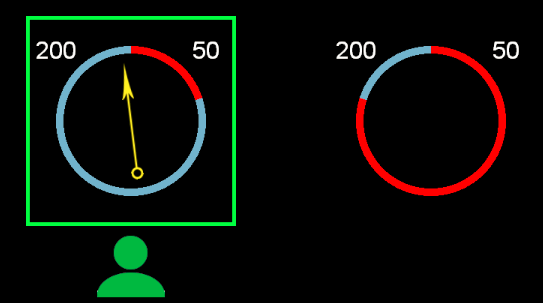


# References

Coricelli, G., Critchley, H. D., Joffily, M., O’Doherty, J. P., Sirigu, A., & Dolan, R. J. (2005). Regret and its avoidance: a neuroimaging study of choice behavior*. Nature Neuroscien*ce*,* 8(9), 1255–1262. https://doi.org/10.1038/nn1514
