# Supplementary material for: Parsing cultural impacts on regret and risk in Iran, China and the United Kingdom
Source: Sci Rep. 2018 Sep 14;8:13862. doi: 10.1038/s41598-018-30680-7 (PMC6138714; doi:10.1038/s41598-018-30680-7)
Supplement: Supplementary file 8 — Supporting Information S7 [file 41598_2018_30680_MOESM8_ESM.docx]

# The files of data and codes:

# Parsing cultural impacts on regret and risk in Iran, China and the United Kingdom

Li Li†, Shiro Kumano†, Anita Keshmirian, Bahador Bahrami* , Jian Li* & Nicholas D. Wright*

* Corresponding Authors (E-mails: bbahrami@gmail.com; li.jian@pku.edu.cn; nick@nicholasdwright.com)

† Both authors contributed equally to the research

1 IranExp1.csv: the dataset of Iranian subjects in Experiment 1.

2 IranExp2.csv: the dataset of Iranian subjects in Experiment 2.

3 ChinaExp1.csv: the dataset of Chinese subjects in Experiment 1.

4 ChinaExp2.csv: the dataset of Chinese subjects in Experiment 2.

5 UKExp1.csv: the dataset of British subjects in Experiment 1.

6 UKExp2.csv: the dataset of British subjects in Experiment 2.

7 DataAnalysis.R: the R code of the data analysis in the study.

8 DataAnalysisFunction.R: the functions used in the code of data analysis.
